# Manganese dioxide mediated one-pot synthesis of methyl 9*H*-pyrido[3,4-*b*]indole-1-carboxylate: Concise synthesis of alangiobussinine

**DOI:** 10.3762/bjoc.7.164

**Published:** 2011-10-12

**Authors:** Jessica Baiget, Sabin Llona-Minguez, Stuart Lang, Simon P MacKay, Colin J Suckling, Oliver B Sutcliffe

**Affiliations:** 1 Strathclyde Institute of Pharmacy and Biomedical Sciences, University of Strathclyde, 165 Cathedral Street, Glasgow, G4 0RE, UK; 2 WestChem, Department of Pure and Applied Chemistry, University of Strathclyde, 295 Cathedral Street, Glasgow, G1 1XL, UK

**Keywords:** alkaloid synthesis, carboline, heterocycle, oxidation, tandem reaction

## Abstract

The carboline ring system is an important pharmacophore found in a number of biologically important targets. Development of synthetic routes for the preparation of these compounds is important in order to prepare a range of analogues containing the carboline heterocyclic moiety. A manganese dioxide mediated one-pot method starting with an activated alcohol and consisting of alcohol oxidation, Pictet–Spengler cyclisation, and oxidative aromatisation, offers a convenient process that allows access to β-carbolines. This one-pot process for the preparation of methyl 9*H*-pyrido[3,4-*b*]indole-1-carboxylate has subsequently been used as the key step in the synthesis of alangiobussinine and a closely related analogue.

## Introduction

Carbolines are an important class of naturally occurring compounds containing the β-carboline motif found in a number of biologically active molecules, and which have recently been shown to be active against Alzheimer’s disease [1–2], bacterial infection [3], inflammation [4–5], HIV and AIDS [6] and various forms of cancers [7–12]. The wide range of therapeutic applications of these molecules highlights the importance of carbolines as a synthetic target in medicinal chemistry. The formation of the β-carboline ring system in nature is well understood and biosynthetically has been shown to proceed by means of either a Pictet–Spengler or a Bischler–Napieralski cyclization followed by an oxidative dehydrogenation process [13–15].

Inspired by nature’s example, we wished to design a synthetic route to the β-carboline scaffold, which was biomimetic and could be carried out in a single operation. One-pot cascade reactions have many advantages over multistep sequences. These include a reduction of the time required to set up the reactions, a removal of the need to isolate unstable intermediates, and a reduction of the time required for purifications. This leads to a lowering of the overall reaction costs and in addition has the advantage that there is less waste associated with reactions of this nature and therefore less environmental impact.

Taylor et al. [16–19] have shown manganese dioxide to be a robust reagent for carrying out oxidations on a wide range of activated alcohols. They have shown that the aldehyde generated does not need to be isolated, but instead can be used in situ allowing further manipulation with a range of suitable reagents, leading to the formation of a variety of different types of molecules as part of a one-pot procedure.

**Scheme 1:**
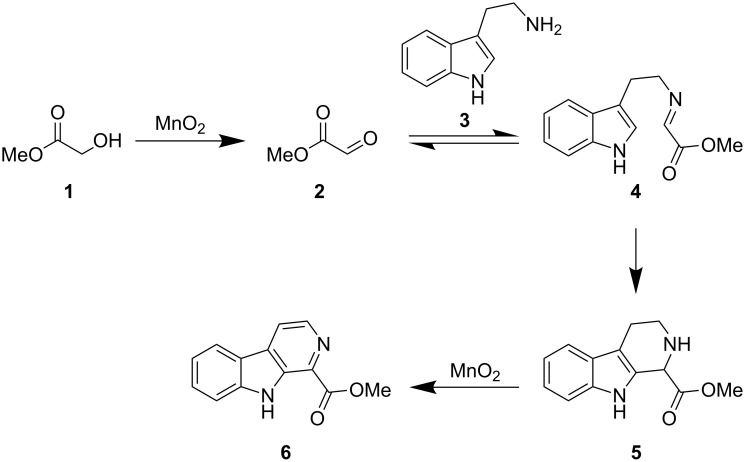
Proposed mechanism for the formation of **6**.

We proposed that exposing methyl glycolate (**1**) to manganese dioxide would result in the formation of aldehyde **2**. This can then undergo a condensation process with tryptamine (**3**) allowing the generation of imine **4**. This intermediate is set up appropriately to undergo a Pictet–Spengler cyclization reaction resulting in the formation of tetrahydrocarboline **5**. In the presence of manganese dioxide intermediate **5** is not isolated, but instead, undergoes a series of dehydrogenation reactions in order to furnish our desired, fully aromatic β-carboline **6** (Scheme 1).

This approach to the synthesis of β-carbolines is particularly elegant as the manganese dioxide has a dual purpose in the reaction. Firstly, it is used for the oxidation of the starting alcohol **1** and secondly it is used in order to promote the aromatisation [20] of tetrahydrocarboline **5**, allowing effectively at least three operational steps to be carried out as part of a single cascade process.

## Results and Discussion

Encouragingly our initial conditions, involving stirring the reaction mixture in toluene at room temperature for 3 h followed by heating under reflux overnight (Table 1, entry a), gave the desired β-carboline **6** in 44% yield as the only product isolated from the reaction mixture. We decided to screen a range of different solvents to assess the optimum conditions in which to carry out this transformation.

**Table 1:** Optimisation of β-carboline **6** synthesis.^a^

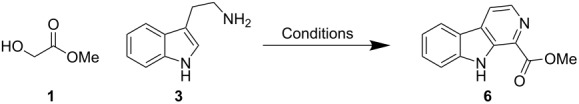			

Entry	Solvent	Additive (equiv)	Yield

a	toluene	none	44%
b	CH_2_Cl_2_	none	0%
c	CHCl_3_	none	39%
d	THF	none	45%
e	DMF^b^	none	26%
f	MeCN	none	51% (35%)^c^
g	1,4-dioxane	none	54% (33%)^c^
h	1,4-dioxane	ZnCl_2_ (1)	0%
i	1,4-dioxane	ZnCl_2_ (0.1)	52%
j	1,4-dioxane	Ti(OiPr)_4_ (0.2)	43%

^a^MnO_2_ (10 equiv), 4 Å molecular sieves, solvent, 3 h at room temperature followed by reflux overnight; ^b^Reaction carried out at 100 °C; ^c^Methyl glycolate (1.5 equiv), MnO_2_ (10 equiv), 4 Å molecular sieves, solvent, 3 h at room temperature followed by heating in the microwave at 170 °C for 2–10 min.

When the reaction was carried out in dichloromethane none of the desired product was formed, and only starting material was detected by both TLC and NMR (Table 1, entry b). Changing the solvent to chloroform showed that chlorinated solvents are compatible in the reaction (Table 1, entry c), and that the low temperature was more detrimental to the process than the nature of the solvent.

The process was found to work best when 1,4-dioxane was used as the solvent, with a yield of 54% being achieved for this one-pot tandem process under these conditions (Table 1, entry g). Disappointingly, although it lowered the time required to carry out the reaction, the use of microwave irradiation led to a lower isolated yield of β-carboline **6** (Table 1, entries f and g), showing that conventional heating is preferred for this process.

We also observed that the use of solvents with lower boiling points, such as dichloromethane, chloroform and THF, did not lead to complete conversion to the desired β-carboline **6**, with both TLC and NMR analysis showing the presence of tryptamine (**3)** in the reaction mixture. We were also unable to detect any of the nonaromatic intermediate **5**, leading us to speculate that the Pictet–Spengler cyclization step, involving conversion of imine **4** to tetrahydrocarboline **5**, is the step with the greatest activation energy in the process.

In an attempt to lower this energy barrier we looked at adding Lewis acids as additives [21] in the process. However, the use of catalytic amounts of Lewis acids did not lead to an increase in the yield of β-carboline **6** (Table 1, entries i and j) and the use of one equivalent of zinc(II) chloride (Table 1, entry h) inhibited all reaction.

This shows that while the use of Lewis acid additives is likely to help with the Pictet–Spengler cyclization step, it may inhibit other steps in the process, e.g., by forming a strong complex with methyl glycolate (**1**) and therefore stopping the manganese dioxide mediated oxidation step. This shows that although Lewis acids can be used to activate Pictet–Spengler reactions, the absence of a Lewis acid is optimum for this one-pot cascade process.

**Figure 1:**
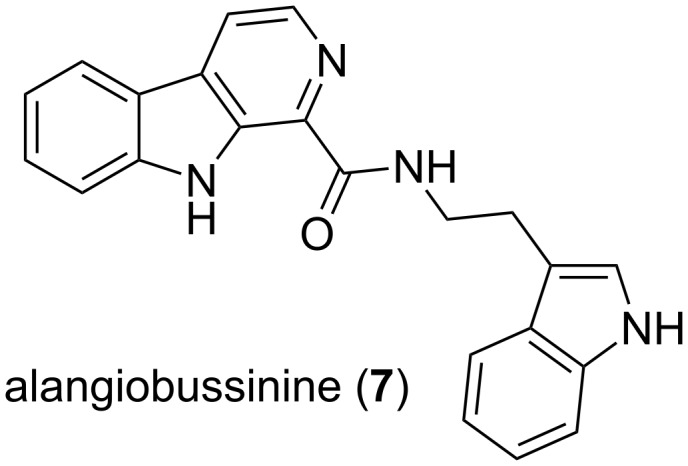
Structure of alangiobussinine (**7**).

β-Carboline **6** is a useful intermediate, used by Panosyan and Still [22] in their synthesis of xestomanzamine A. We wished to apply the preparation of β-carboline **6** as the key synthetic step in the first reported synthesis of alangiobussinine, although the synthesis of dihydroalangiobussinine [23] has been reported in low yield as a byproduct by Laronze and coworkers. Alangiobussinine (**7**, Figure 1) is an alkaloid that is isolated from the leaves of Alangium bussyanum, a tropical shrub.

**Scheme 2:**
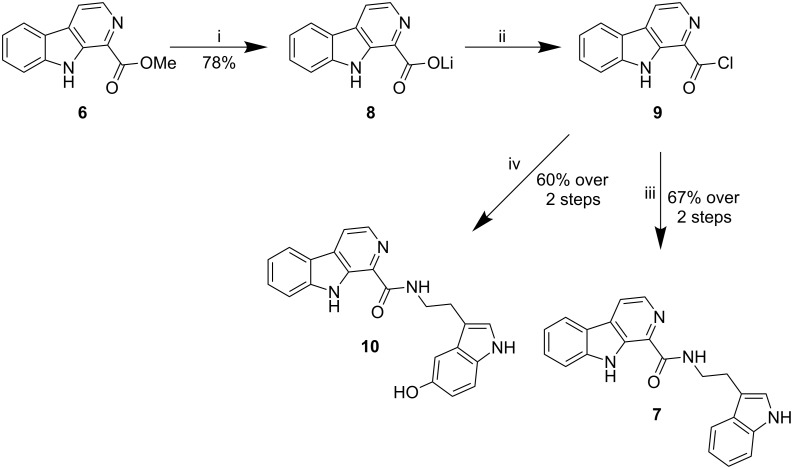
Preparation of compounds **7** and **10**. Reagents and conditions: i) LiOH (10 equiv), MeOH–H_2_O, rt, overnight; ii) oxalyl chloride (5 equiv), DMF (0.01 equiv), CH_2_Cl_2_, rt, 6 h; iii) tryptamine (**3)** (2 equiv), Et_3_N (3 equiv), MeCN, 0 °C to rt, overnight; iv) serotonine·HCl (2 equiv), Et_3_N (3 equiv), MeCN, 0 °C to rt, overnight.

Our strategy for the completion of the synthesis of alangiobussinine (**7**) involved initial hydrolysis of carboline ester **6** with lithium hydroxide in a mixture of methanol and water. The reaction mixture was then partitioned between dichloromethane and water with the lithium carboxylate **8** being insoluble in both layers, but precipitating exclusively in the dichloromethane layer. Lithium carboxylate **8** was therefore isolated in 78% yield by separation of the two layers followed by filtration, and could be used directly in the next step without need for an additional protonation step. Lithium carboxylate **8** was then converted to acid chloride **9**, which was isolated and treated directly with tryptamine (**3**) to give access to alangiobussinine (**7**) in 67% yield over the two steps (Scheme 2), with our spectroscopic data being consistent with that of the natural product [24].

In addition to preparing alangiobussinine (**7**), we also used the same strategy to prepare its structural analogue **10**, which was achieved in a combined yield of 60% for both the acid-chloride formation and amide-bond-formation steps. It was also possible to use T3P^®^, along with Et_3_N, in order to couple lithium carboxylate **8** with both tryptamine (**3**) and serotonin. This one-step process led to the formation of the desired compounds **7** and **10**, although disappointingly only in 19% and 24% yield, respectively, and therefore offers no synthetic benefits over the two-step protocol.

In summary, functionalised β-carboline **6** has been synthesised by a manganese dioxide mediated one-pot oxidation, condensation, Pictet–Spengler cyclization, dehydrogenative-aromatisation cascade process. This procedure was then applied as the key step in our divergent synthetic strategy for formation of the naturally occurring alkaloid, alangiobussinine (**7**) and its analogue **10**.

## Supporting Information

File 1 Experimental section.
