# Supplementary material for: Manganese dioxide mediated one-pot synthesis of methyl 9H-pyrido[3,4-b]indole-1-carboxylate: Concise synthesis of alangiobussinine
Source: Beilstein J Org Chem. 2011 Oct 12;7:1407–11. doi: 10.3762/bjoc.7.164 (PMC3201054; doi:10.3762/bjoc.7.164)
Supplement: File 1 — Experimental section. [file Beilstein_J_Org_Chem-07-1407-s001.pdf]

# Supporting Information

for

## Manganese dioxide mediated one-pot synthesis of methyl 9*H*-pyrido[3,4-*b*]indole-1-carboxylate: Concise synthesis of alangiobussinine

Jessica Baiget[1], Sabin Llona-Minguez[1], Stuart Lang[2*], Simon P. MacKay[1],
Colin J. Suckling[2] and Oliver B. Sutcliffe[1]

Address: [1]Strathclyde Institute of Pharmacy and Biomedical Sciences, University of Strathclyde, 165 Cathedral Street, Glasgow, G4 0RE, UK and [2]WestChem, Department of Pure and Applied Chemistry, University of Strathclyde, 295 Cathedral Street, Glasgow, G1 1XL, UK

Email: Stuart Lang[*] - stuart.lang@strath.ac.uk

[*] Corresponding author

## Experimental section

Starting materials were obtained from commercial sources and used as received. Purification of final compounds was achieved by means of flash chromatography or Biotage SP4 columns. $^{1}$H NMR spectra were recorded with DMSO-$d_6$ at 400 MHz and $^{13}$C NMR spectra were recorded at 100 MHz on a JOEL 400 NMR instrument. FTIR spectra were obtained on a Thermo Scientific Nicolet iS10 and melting points were measured on a Stuart Scientific SMP1. HRMS spectra were recorded on a Thermo Scientific Exactive Orbitrap Mass Analyzer.

**Methyl 9*H*-pyrido[3,4-*b*]indole-1-carboxylate (6)**

**Table 1, entry a**

Tryptamine (1 g, 6.24 mmol), methyl glycolate (0.72 mL, 9.36 mmol), 4 Å molecular sieves (6.24 g) and $MnO_2$ (8.14 g, 93.6 mmol) were stirred at room temperature in

toluene (65 mL) for 3 hours. The mixture was then heated under reflux overnight. The mixture was cooled and the solids were removed by filtration through Celite® and washed with ethyl acetate (100 mL). The solvent was removed and the residue was purified by flash chromatography eluting with 5% methanol in dichloromethane to give β-carboline **6** (620 mg, 44%) as cream-coloured powder.

**Table 1, entry b**

Methyl glycolate (232 μL, 3 mmol) was added to a suspension of tryptamine (321 mg, 2 mmol), $MnO_2$ (1.7 g, 20 mmol) and 4 Å molecular sieves (2 g) in dichloromethane (20 mL). The mixture was stirred at room temperature for 3 hours under nitrogen. The mixture was then heated under reflux overnight. The mixture was cooled and the solids were removed by filtration through Celite® and washed with ethyl acetate (30 mL). The filtrates were concentrated in vacuo and dried. The $^1$H NMR of the crude mixture did not show any product.

**Table 1, entry c**

Methyl glycolate (232 μL, 3 mmol) was added to a suspension of tryptamine (320 mg, 2.0 mmol), $MnO_2$ (1.7 g, 20 mmol) and 4 Å molecular sieves (2 g) in chloroform (20 mL). The mixture was stirred at room temperature for 3 hours under nitrogen. The mixture was then heated under reflux overnight. The mixture was cooled and the solids were removed by filtration through Celite® and washed with ethyl acetate (30 mL). The filtrates were concentrated in vacuo and dry loaded to a 10 g Biotage SP4 cartridge for chromatographic purification (solvent system: Hexane/ethyl acetate, gradient: 0% 4CV (cartridge volume, which for a 10 g Biotage SP4 cartridge is 20 mL); 0–30% 10CV; 30% 6CV) to give β-carboline **6** (175 mg, 39%).

**Table 1, entry d**

Tryptamine (1 g, 6.24 mmol), methyl glycolate (0.72 mL, 9.36 mmol) and 4 Å molecular sieves (6.24 g) and $MnO_2$ (8.14 g, 93.6 mmol) were stirred at room temperature in THF (65 mL) for 3 hours. The mixture was then heated under reflux overnight. The mixture was cooled and the solids were removed by filtration through Celite® and washed with ethyl acetate (100 mL). The solvent was removed and the residue purified by flash chromatography eluting with 5% methanol in dichloromethane to give β-carboline **6** (633 mg, 45%).

**Table 1, entry e**

Methyl glycolate (232 μL, 3 mmol) was added to a suspension of tryptamine (320 mg, 2.0 mmol), $MnO_2$ (1.7 g, 20 mmol) and 4 Å molecular sieves (2 g) in DMF (20 mL).

The mixture was stirred at room temperature for 3 hours under nitrogen. The mixture was then heated at 100 °C overnight. The mixture was cooled and the solids were removed by filtration through Celite® and washed with ethyl acetate (30 mL). The filtrates were concentrated in vacuo and dry loaded to a 10 g Biotage SP4 cartridge for chromatographic purification (solvent system: Hexane/ethyl acetate, gradient: 0% 4CV; 0–30% 10CV; 30% 6CV) to give β-carboline **6** (119 mg, 26%).

**Table 1, entry f (conventional heating)**

Methyl glycolate (232 μL, 3 mmol) was added to a suspension of tryptamine (320 mg, 2 mmol), $MnO_2$ (1.7 g, 20 mmol) and 4 Å molecular sieves (2 g) in MeCN (20 mL). The mixture was stirred at room temperature for 3 hours under nitrogen. The mixture was then heated under reflux overnight. The mixture was cooled and the solids were removed by filtration through Celite® and washed with ethyl acetate (30 mL). The filtrates were concentrated in vacuo and dry loaded to a 10 g Biotage SP4 cartridge for chromatographic purification (solvent system: Hexane/ethyl acetate, gradient: 0% 4CV; 0–30% 10CV; 30% 6CV) to give β-carboline **6** (230 mg, 51%).

**Table 1, entry f (microwave heating)**

Methyl glycolate (237 μL, 3.13 mmol) was added to a suspension of tryptamine (334 mg, 2.08 mmol), $MnO_2$ (1.7 g, 20 mmol) and 4 Å molecular sieves (2 g) in MeCN (12 mL). The mixture was stirred at room temperature for 3 hours under nitrogen. The mixture was then subjected to microwave irradiation at 170 °C for 2 minutes. The mixture was cooled and the solids were removed by filtration through Celite® and washed with ethyl acetate (30 mL). The filtrates were concentrated in vacuo and dry loaded to a 10 g Biotage SP4 cartridge for chromatographic purification (solvent system: Hexane/ethyl acetate, gradient: 0% 4CV; 0–30% 10CV; 30% 6CV) to give β-carboline **6** (163 mg, 35%).

**Table 1, entry g (conventional heating)**

Methyl glycolate (232 μL, 3 mmol) was added to a suspension of tryptamine (320 mg, 2 mmol), $MnO_2$ (1.7 g, 20 mmol) and 4 Å molecular sieves (2 g) in 1,4-dioxane (20 mL). The mixture was stirred at room temperature for 3 hours under nitrogen. The mixture was then heated under reflux overnight. The mixture was cooled and the solids were removed by filtration through Celite® and washed with ethyl acetate (30 mL). The filtrates were concentrated in vacuo and dry loaded to a 10 g Biotage SP4 cartridge for chromatographic purification (solvent system: Hexane/ethyl acetate, gradient: 0% 4CV; 0–30% 10CV; 30% 6CV) to give β-carboline **6** (247 mg, 54%).

**Table 1, entry f (microwave heating)**

Methyl glycolate (232 μL, 3 mmol) was added to a suspension of tryptamine (320 mg, 2 mmol), $MnO_2$ (1.7 g, 20 mmol), 4 Å molecular sieves (2 g) in 1,4-dioxane (12 mL). The mixture was stirred at room temperature for 3 hours under nitrogen. The mixture was then subjected to microwave irradiation at 170 °C for 10 minutes. The mixture was cooled and the solids were removed by filtration through Celite® and washed with ethyl acetate (30 mL). The filtrates were concentrated in vacuo and dry loaded to a 10 g Biotage SP4 cartridge for chromatographic purification (solvent system: Hexane/ethyl acetate, gradient: 0% 4CV; 0–30% 10CV; 30% 6CV) to give β-carboline **6** (148 mg, 33%).

**Table 1, entry h**

Methyl glycolate (232 μL, 3 mmol) was added to a suspension of tryptamine (320 mg, 2 mmol), $MnO_2$ (1.7 g, 20 mmol), the 4 Å molecular sieves (2 g) and $ZnCl_2$ (273 mg, 2 mmol) in 1,4-dioxane (20 mL). The mixture was stirred at room temperature for 3 hours under nitrogen. The mixture was then heated under reflux overnight. The mixture was cooled and the solids were removed by filtration through Celite® and washed with ethyl acetate (30 mL). The filtrates were concentrated in vacuo. The NMR of the crude mixture did not show any product.

**Table 1, entry i**

Methyl glycolate (232 μL, 3 mmol) was added to a suspension of tryptamine (320 mg, 2 mmol), $MnO_2$ (1.7 g, 20 mmol), the molecular sieves (2 g) and $ZnCl_2$ (28 mg, 0.2 mmol) in 1,4-dioxane (20 mL). The mixture was stirred at room temperature for 3 hours under nitrogen. The mixture was then heated under reflux overnight. The mixture was cooled and the solids were removed by filtration through Celite® and washed with ethyl acetate (30 mL). The filtrates were concentrated in vacuo and dry loaded to a 10 g Biotage SP4 cartridge for chromatographic purification (solvent system: Hexane/ethyl acetate, gradient: 0% 4CV; 0–30% 10CV; 30% 6CV) to give β-carboline **6** (235 mg, 52%). mp 163–164 °C, lit [1] 165–166 °C; HRMS (ESI) calcd for $C_{13}H_{11}N_2O_2$ $[M + H]^+$ 227.0821, found: 227.0815; FTIR (neat): 1675, 1315, 728 $cm^{-1}$; $^1H$ NMR (400 MHz, DMSO-$d_6$) δ: 11.64 (s, 1H), 8.48 (d, *J* = 4.9 Hz, 1H), 8.41 (d, *J* = 4.9 Hz, 1H), 8.30 (d, *J* = 7.8 Hz, 1H), 7.78 (d, *J* = 8.2 Hz, 1H), 7.62 (dd, *J* = 8.2 and 7.1 Hz, 1H), 7.32–7.29 (m, 1H) 4.02 (s, 3H); $^{13}C$ NMR (100 MHz, DMSO-$d_6$) δ: 165.7, 141.3, 137.8, 135.8, 130.7, 129.6, 128.9, 121.7, 119.9, 118.7, 112.7, 99.4, 52.0.

**Table 1, entry j**

Methyl glycolate (232 μL, 3 mmol) was added to a suspension of tryptamine (320 mg, 2 mmol), $MnO_2$ (1.7 g, 20 mmol), 4 Å molecular sieves (2 g) and $Ti(OiPr)_4$ (117 μl, 0.4 mmol) in 1,4-dioxane (20 mL). The mixture was stirred at room temperature for 3 hours under nitrogen. The mixture was then heated under reflux overnight. The mixture was cooled and the solids were removed by filtration through Celite® and washed with ethyl acetate (30 mL). The filtrates were concentrated in vacuo and dry loaded to a 10 g Biotage SP4 cartridge for chromatographic purification (solvent system: Hexane/ethyl acetate, gradient: 0% 4CV; 0–30% 10CV; 30% 6CV) to give β-carboline **6** (193 mg, 43%).

**Lithium 9*H*-pyrido[3,4-*b*]indole-1-carboxylate (8)**

Methyl 9*H*-pyrido[3,4-*b*]indole-1-carboxylate (1 g, 4.44 mmol) was suspended in methanol (20 mL) and water (20 mL), and lithium hydroxide (1.06 g, 44.4 mmol) was added. The mixture was stirred overnight. Water (200 mL) was added and this was extracted with dichloromethane (4 × 100 mL). The organic layer was filtered and the solid was collected to give carboxylate **8** (750 mg, 78%) as a white powder. mp >360 °C; HRMS (ESI) calcd for $C_{12}H_9N_2O_2$ $[M + H]^+$: 213.0659, found: 213.0659; FTIR (neat): 3325, 1614, 727 $cm^{-1}$; $^1H$ NMR (400 MHz, DMSO-$d_6$ + 1 drop 35% DCl in $D_2O$) δ: 9.00 (d, $J$ = 5.9 Hz, 1H), 8.61 (d, $J$ = 5.9 Hz, 1H), 8.5 (d, $J$ = 8.0 Hz, 1H), 7.91 (d, $J$ = 8.3 Hz, 1H), 7.78–7.82 (m, 1H), 7.42–7.46 (m, 1H); $^{13}C$ NMR (100 MHz, DMSO-$d_6$ + 1 drop 35% DCl in $D_2O$) δ: 161.7, 145.2, 137.7, 134.6, 133.2, 131.1, 124.3, 123.6, 122.6, 120.7, 119.5, 114.2.

**Alangiobussinine, *N*-(2-(1*H*-indol-3-yl)ethyl)-9*H*-pyrido[3,4-*b*]indole-1-carboxamide (7)**

**Method A**

DMF (0.43 μL, 5.5 nmol) was added to a stirred suspension of lithium 9*H*-pyrido[3,4-*b*]indole-1-carboxylate (120 mg, 0.55 mmol) and oxalyl chloride (0.24 mL, 349 mg, 2.75 mmol) in dry dichloromethane (10 mL). The mixture was refluxed for 6 hours (TLC of sample after addition to methanol showed total conversion to methyl ester; acid chloride hydrolyses on TLC to acid). Toluene (10 mL) was added and all the volatiles were distilled off in vacuo, yielding the acid chloride as an orange powder, which was immediately used in the next step without further purification. The residue was suspended in dry MeCN (20 mL) and added by syringe to a stirred suspension of

tryptamine (240 mg, 1.49 mmol) and dry $Et_3N$ (0.31 mL, 227 mg, 2.24 mmol) in dry MeCN (10 mL) at 0 °C. The reaction was stirred at room temperature overnight, subsequently concentrated in vacuo and the residue was triturated with $H_2O$ (1 mL) (10 g Biotage SP4 cartridge solvent system: MeOH/$CH_2Cl_2$, gradient: 0% 6CV; 0–10% 12CV) to yield the desired compound **7** (131 mg, 67%) as a cream-coloured powder.

**Method B**

A 50+% solution in DMF of 1-propylphosphonic acid cyclic anhydride (T3P®) (0.18 mml, 263 mg, 0.83 mmol) was added under $N_2$ atmosphere to a stirred suspension of lithium 9*H*-pyrido[3,4-*b*]indole-1-carboxylate (**8**) (120 mg, 0.55 mmol) and tryptamine (106 mg, 0.66 mmol) in dry THF (8 mL). The mixture was stirred at room temperature for 60 hours. THF was subsequently distilled off in vacuo, saturated aqueous $NaHCO_3$ (10 mL) was added to the residue and the aqueous layer was washed with EtOAc (3 × 10 mL). The organic layer was dried over anhydrous $MgSO_4$, concentrated in vacuo and dry loaded to a 10 g Biotage SP4 cartridge for chromatographic purification (solvent system: MeOH/$CH_2Cl_2$, gradient: 0% 6CV; 0–10% 12CV), which gave the desired compound **7** (37 mg, 19%) as a cream-coloured powder [2]. mp 202–204 °C; HRMS (ESI) calcd for $C_{22}H_{18}N_4O$ $[M + H]^+$: 355.1553, found: 355.1554; FTIR (neat): 3441, 3373, 3266, 1645, 1645, 1528, 738, 729 $cm^{-1}$; $^1H$ NMR (400 MHz, DMSO-$d_6$) δ: 11.78 (bs, 1H), 10.84 (bs, 1H), 9.07 (t, $J$ = 6.0 Hz, 1H), 8.39 (d, $J$ = 5.0 Hz, 1H), 8.35 (d, $J$ = 5.0 Hz, 1H), 8.28 (d, $J$ = 7.9 Hz, 1H), 7.81 (d, $J$ = 8.2 Hz, 1H), 7.67 (d, 7.8 Hz, 1H), 7.56–7.59 (m, 1H), 7.35 (d, $J$ = 8.0 Hz, 1H), 7.26–7.29 (m, 1H), 7.23 (d, $J$ = 2.12 Hz, 1H), 7.06–7.10 (m, 1H), 6.98–7.02 (m, 1H), 3.71 (td, $J$ = 7.5 and 6.0 Hz, 2H), 3.04 (t, $J$ = 7.5 Hz, 2H); $^{13}C$ NMR (100 MHz, DMSO-$d_6$) δ: 166.0, 142.2, 137.2, 136.9, 135.0, 133.2, 131.2, 129.3, 127.8, 123.2, 122.3, 121.6, 120.4, 120.3, 119.0, 118.8, 118.4, 113.6, 112.4, 112.0, 40.0 26.0.

***N*-(2-(5-hydroxy-1*H*-indol-3-yl)ethyl)-9*H*-pyrido[3,4-*b*]indole-1-carboxamide (10)**

**Method A**

DMF (0.53 µL, 6.7 nmol) was added to a stirred suspension of lithium 9*H*-pyrido[3,4-*b*]indole-1-carboxylate (150 mg, 0.69 mmol) and oxalyl chloride (0.30 mL, 3.44 mmol) in dry dichloromethane (10 mL). The mixture was heated under reflux for 6 hours (TLC of sample after addition to methanol showed total conversion to methyl ester; acid chloride hydrolyses on TLC to acid), toluene (10 mL) was added and all the volatiles were distilled off in vacuo, yielding the acid chloride as an orange

powder, which was immediately used in the next step without further purification. The residue was suspended in dry acetonitrile (20 mL) and added by syringe to a stirred suspension of serotonine hydrochloride (293 mg, 1.38 mmol) and dry $NEt_3$ (0.29 mL, 2.06 mmol) in dry acetonitrile (10 mL) at 0 °C. The reaction mixture was stirred at room temperature overnight, subsequently concentrated in vacuo and the residue triturated with distilled water (2 mL) and dry loaded to a 10 g Biotage SP4 cartridge for chromatographic purification (solvent system: Methanol/dichloromethane, gradient: 0% 6CV; 0–10% 10CV; 10% 6CV). Concentration in vacuo of fractions containing the product yielded an orange gum which was subsequently triturated with methanol (0.2 mL) and diethyl ether (1 mL) to yield compound **10** (152 mg, 60%) as a yellow solid.

**Method B**

A 50+% solution in DMF of 1-propylphosphonic acid cyclic anhydride (0.18 mL, 0.83 mmol) was added under $N_2$ atmosphere to a stirred suspension of lithium 9*H*-pyrido[3,4-*b*]indole-1-carboxylate (120 mg, 0.55 mmol) and serotonin hydrochloride (140 mg, 0.66 mmol) in dry THF (8 mL). The mixture was stirred at room temperature for 60 hours. THF was subsequently distilled off in vacuo, saturated aqueous $NaHCO_3$ (10 mL) was added to the residue and the aqueous layer was washed with EtOAc (3 × 10 mL). The organic layer was dried over anhydrous $MgSO_4$, concentrated in vacuo and dry loaded to a 10 g Biotage SP4 cartridge for chromatographic purification (Solvent system: MeOH/DCM, gradient: 0% 6CV; 0–10% 10CV; 10% 6CV). Concentration in vacuo of fractions containing the product yielded an orange gum, which was subsequently triturated with MeOH (0.5 mL) and diethyl ether (1 mL) to yield the product **10** (49 mg, 24%) as a yellow solid. mp 234–236 °C; HRMS (ESI) calcd for $C_{22}H_{18}N_4O_2$ $[M + H]^+$: 371.1503, found: 371.1499; FTIR (neat): 3443, 3375, 3240, 1645, 1620, 1529, 757, 729 $cm^{-1}$; $^1H$ NMR (400 MHz, DMSO-$d_6$) δ: 11.79 (bs, 1H), 10.52 (bs, 1H), 9.03 (t, *J* = 6.0 Hz, 1H), 8.61 (bs, 1H) 8.39 (d, *J* = 5.0 Hz, 1H), 8.35 (d, *J* = 5.0 Hz, 1H), 8.28 (d, *J* = 7.9 Hz, 1H), 7.80 (d, *J* = 8.2 Hz, 1H), 7.57–7.58 (m, 1H), 7.26–7.27 (m, 1H), 7.12–7.13 (m, 2H), 6.95 (d, *J* = 2.1 Hz, 1H), 6.61 (dd, *J* = 8.6 and 2.1 Hz, 1H), 3.71 (dt, *J* = 7.5 and 6.0 Hz, 2H), 2.95 (t, *J* = 7.5 Hz, 2H); $^{13}C$ NMR (100 MHz, DMSO-$d_6$) δ: 165.9, 150.8, 142.2, 137.1, 134.9, 133.2, 131.5, 131.2, 129.3, 128.5, 123.7, 122.3, 120.4, 120.3, 118.4, 113.6, 112.2, 111.9, 111.4, 103.0, 39.8, 26.1.

## References

1. Srinivasan, N.; Ganesan, A. *J. Chem. Soc., Chem. Commun.* **2003,** 916–917. doi:10.1039/b212063a
2. Panosyan, F. B.; Still, I. W. J. *Can. J. Chem.* **2001,** *79*, 1110–1114. doi:10.1139/v01-092
